# Evaluation of Factors Contributing to Excessive Nitrate Accumulation in Fodder Crops Leading to Ill-Health in Dairy Animals

**DOI:** 10.4103/0971-6580.75848

**Published:** 2011

**Authors:** P. K. Sidhu, G. K. Bedi, Meenakshi, V. Mahajan, S. Sharma, K. S. Sandhu, M. P. Gupta

**Affiliations:** Department of Epidemiology and Preventive Veterinary Medicine, College of Veterinary Science, Guru Angad Dev Veterinary and Animal Sciences, University, Ludhiana, Punjab, India

**Keywords:** Dairy animals, fertilizers, fodder crops, nitrate

## Abstract

A study was conducted to estimate nitrate content in commonly used fodder crops, viz., berseem (*Trifolium alexandrinum*), bajra (*Pennisetum glaucum*), maize (*Zea mays*), oats (*Avena sativa*), sorghum (*Sorghum vulgare*) and toriya (*Brassica napus*), collected from the fields of different villages of Punjab and farms of the university, and to evaluate the factors associated with nitrate accumulation in these crops. The nitrate level was highest in sorghum on dry matter basis, followed by oats and toriya, berseem, maize and bajra. The nitrate content was also determined in fodder samples harvested from young and mature stages and in different parts of plants. The stem part of forages had higher content than leaves; however, concentrations were low in mature crops as compared to immature ones. The environmental and soil factors associated with it are discussed and correlated with the experimental findings.

## INTRODUCTION

Dairy cows fed on high-quality forage produce more milk with less supplemental concentrate than the cows fed lower-quality forages. Forages with high concentrations of crude protein (CP) are considered high quality because feeding high-protein forage cuts down the need of supplemental protein. Secondly, CP content is positively correlated to energy content of forages. High-protein forages generally are more digestible and provide more energy than low-protein forages. Application of nitrogen (N) fertilizer improves both quality and yield by increasing CP content of forage markedly and the available energy. Due to this reason, the use of N-fertilizers on agricultural land has been immensely increased around the world.[1]

Forages take up and assimilate nitrogen as NH_4_^+^, NO_3_^-^ and soluble organic compounds such as urea (CO(NH_2_)_2_) and amino-acids.[2] Nitrate is the primary nutrient form of the nitrogen in soils and is a normal constituent of plants. Occasionally, excessive amounts of nitrate accumulate in plants and result in livestock mortalities. Outbreaks of nitrate toxicity due to consumption of fodder containing high amounts of nitrate have occurred in farm animals throughout the world.[3] Most commonly, nitrate poisoning occurs in cattle and sheep. In ruminants, nitrate is reduced by microbial reductases to nitrite. The rumen microbes utilize this nitrite by converting it into ammonia as a nitrogenous source. However, excessive nitrite gets accumulated in rumen, from where it is readily absorbed into blood stream and combines with ferrous ion of hemoglobin (Hb) to form met-hemoglobin (met-Hb). The met-Hb is a poor transporter of oxygen in the body and the animal suffers from oxygen deficiency.

In livestock, poisoning due to nitrate ions is influenced by several factors that include plant, environmental, management factors and health status of the animal. The plant factors are the most important amongst these because nitrate toxicity in livestock is chiefly caused by consumption of plants rich in nitrates. The factors that influence the accumulation of nitrate in fodder crops are species, stage of growth, part of plant, pH of soil, use of fertilizers and climatic conditions. These factors had not been studied recently and the guidelines found today in literature are based on limited research data obtained in the 60s and 70s, and have not been updated to more recent research and field experiences. Keeping this in view, factors contributing toward accumulation of nitrate in forages were studied and correlated with recent research and the field problems encountered in dairy animals due to excessive exposure to nitrate rich plants.

## MATERIALS AND METHODS

Six forage crops, viz., berseem (*Trifolium alexandrinum*), oats (*Avena sativa*), sorghum (*Sorghum vulgare*), bajra (*Pennisetum typhoides*), toriya (*Brassica napus*), and maize (*Zea mays*), commonly used as fodder for farm animals, were evaluated for the nitrate content and the factors associated with toxicity and its accumulation were studied.

The nitrate content was estimated in samples of bajra, berseem, maize, oats, sorghum and toriya that were collected randomly from different villages of five districts (Ludhiana, Moga, Jalandhar, Patiala and Sangrur) of Punjab state. Samples were also collected from fields of the Punjab Agricultural University, where fodder was grown without the use of chemical fertilizers. Nitrate estimations were done separately as whole plant, stem and leaves of plants. Samples of guinea grass were collected from different cuttings (first to fourth cut) from fields and the nitrate content was estimated. The fodder samples received in the toxicology laboratory from farmers of Punjab state, suspected to be contaminated, were also tested for nitrate levels and their background history was collected.

The fodder samples were initially subjected to qualitative test with diphenylamine blue (DPB-1% in concentrated sulfuric acid) to know the presence/absence of nitrate.[4] The same fodder samples were finally subjected to quantitative estimation of nitrates as per the method described by Cataldo *et al*,.[5]

## RESULTS AND DISCUSSION

The amount of nitrate accumulated within the plant depends upon the rate of nitrogen uptake by the plant from the soil and the rate of its reduction by the plant. There is no accumulation when the rate of reduction equals the rate of uptake and when uptake exceeds the rate of reduction, nitrate starts getting accumulated. Some species, viz., sorghum, oats, sudan grass, etc., are known nitrate accumulators and may cause sudden death in animals.[6] Nitrate can be detected in traceable amounts in all plants but it becomes dangerous when it exceeds the safe limit of 2500 ppm NO_3_-N and forages having more than 4500 ppm NO_3_-N are considered highly toxic. Seven outbreaks due to consumption of fodder having excessive amount of nitrate have been recorded in bovines in Punjab during 2002–2007 (Annual Progress Reports, Animal Disease Research Centre, GADVASU, Ludhiana; 2002-2007; unpublished data). Two dreadful outbreaks associated with consumption of fodder containing toxic amount of nitrate occurred recently in Tajpur and Haibowal Dairy Complexes, Ludhiana, Punjab, and 105 dairy cattle died in these unfortunate incidents. Even the low level of nitrate in fodder may lead to slight respiratory distress and help respiratory infections to flourish. Reduced weight gain and impaired fertility has been reported in cattle grazing pasture having NO_3_-N in the range of 801–3400 ppm.[7] Therefore, six forage crops commonly used as fodder for farm animals in India were evaluated for nitrate content and the factors influencing its accumulation were studied.

The mean values of nitrate content determined in common fodder crops collected from villages of Punjab are presented in Table 1. The nitrate content on dry matter basis was highest in sorghum (1695 ppm), followed by oats (1525 ppm) and toriya (1510 ppm). The nitrate levels in maize, berseem and bajra were 825, 990 and 776 ppm, respectively. The study revealed that oats, sorghum and toriya are the natural accumulators of nitrate. In previous literature, it was found that sorghum is one of the most notorious accumulators of nitrate but no reference suggesting excessive amounts of nitrogen in toriya could be traced.[3] There was no environmental stress when these samples were collected. The reason could be the presence of excess soil nitrogen than that required for maximum growth of oats, sorghum and toriya. In Punjab, farmers have been using nitrate containing fertilizers (urea, ammonium nitrate) in their fields for 30 years without following any fertility program, to obtain higher yields of crops. Therefore, it is assumed that in general, the farming soil might be rich in nitrogen in the state. This needs to be investigated further.

**Table 1:** Mean nitrate content in fodder samples (whole plant) collected from villages of Punjab state and farms of Punjab Agricultural University (PAU), Ludhiana

Crop	Number of samples (*n*)	Village sample* NO_3_-N (ppm)	PAU farm samples* NO_3_-N (ppm)	PAU farm samples** NO_3_-N (ppm)
		Mean (range)	Mean (range)	Mean (range)
Oats	65	1525 (985–2035)	935 (850–1075)	1480 (1260–1855)
Sorghum	30	1695 (1025–2240)	1080 (805–1250)	NA
Berseem	82	990 (650–1970)	675 (510–945)	1056 (805–1265)
Bajra	30	776 (450–775)	565 (524–762)	760 (574–795)***
Maize	50	825 (610–1234)	515 (505–670)	745 (610–940)
Toriya	50	1510 (820–1950)	1220 (805–1455)	1670 (1150–1852)

* Samples collected during normal days;

** Samples collected during harsh winter days and cloudy environment;

*** Samples collected during dry, very hot and humid weather;

NA – Data not available

It was interesting to note that oats and toriya grown together with barseem in the same field were found to contain two times of nitrate compared to barseem. It indicates the intrinsic ability of oat and toriya to accumulate nitrate [Table 2]. Two outbreaks of nitrate poisoning that occurred recently in Ludhiana were the result of huge consumption of toriya alone by hungry cattle and buffaloes, which were not previously fed this fodder (data not shown). The nitrate level of toriya on dry matter basis was 5200–5500 ppm at Tajpur Dairy Complex outbreak and 6820–7040 ppm at Haibowal Dairy Complex outbreak. At Haibowal Dairy Complex, the fodder comprised barseem + toriya. The fodder was grown together in the same field and barseem contained nitrate of 800 ppm. This suggested the inherent ability of toriya to accumulate nitrate. Previous literature supports the differences in potential for nitrate accumulation among the species and varieties of forages.[4] Another consistent finding was higher nitrate content in radish than in berseem, oats and toriya samples despite being grown together in the same field. The nitrate concentration in radish was 5–6 times higher than barseem and 2-3 times more when compared to oats and toriya. It is confirmed that nitrate accumulation in fodder may vary depending upon the genetic variability in nitrate uptake by a plant. The farmers should select particular forage for feeding of livestock keeping in mind the nitrate content of forages and crops available at their disposal.

**Table 2:** Nitrate content in fodder samples grown as mixed crops in the same field (mean value, *n* = 30)

Crop combination	NO_3_-N (ppm)			
	Berseem	Oats	Toriya	Radish
Barseem + oats + radish	704	1675	NA	Root = 3500 Leaves = 1145
Barseem + toriya + radish	876	NA	1425	Root = 5000 Leaves = 1075
Barseem + oats + toriya + radish	825	1594	1500	Root = 4700 Leaves = 1267

NA – Data not available

Nitrate accumulation varies with the stage of plant growth. Rate of uptake diminishes with the maturity of the plant. Therefore, immature (young) crop contain more nitrate than the mature crop.[3] The increased frequency of occurrence of outbreaks in farm animals due to nitrate poisoning in the months of December and January every year may be correlated with this factor (data not shown). In these extreme winter months, young crops rich in nitrate are fed due to scarcity of green fodder. This was reflected in the recent outbreaks of Tajpur and Haibowal Dairy Complexes, where feeding young toriya took the lives of 105 dairy cattle.

To determine the effect of age of plant on nitrate levels, berseem, bajra, maize, oats and toriya were obtained at two levels of cuttings (mature and immature) from the same fields to determine the nitrate content. The nitrate level reduced in all forages with the age of plant. The difference in nitrate level of mature and immature forages was statistically significant at 5% level of significance [Table 3]. The effect of plant maturity yielded interesting results in maize and bajra where nitrate

concentrated - double in stem than in leaves. It showed that distribution of nitrate varies with the age of plant. It might be due to the distribution of some nitrate in the grains of maize and bajra which appear at the mature stage. The nitrate level was quite high in samples of young crops of oats, toriya and sorghum received from farmers in the laboratory for testing as compared to mature samples of these crops (young crops NO_3_-N = 1568-2054 ppm; mature crops NO_3_-N = 972-1232 ppm). The results confirmed the previous reports of reduced nitrate levels with the maturity of plant.[8] Lower nitrate levels in mature plant may be due to decreased uptake or increased enzyme activity to convert the nitrate into intermediate compounds ready for evaporation or used by the plant. It is suggested to delay the harvesting of known nitrate accumulators (toriya, oats and sorghum) for reducing the dangerous nitrate levels.

**Table 3:** Distribution of nitrate content in fodder samples collected from Punjab Agricultural University farms (mean value; *n* = 30)

	NO_3_-N (ppm)		
		Young plant	Mature plant
Barseem	Leaves	615	430
	Stem	835	576
	Whole plant	745	505
Oats	Leaves	1195	965
	Stem	1465	1170
	Whole plant	1275	1005
Toriya	Leaves	1024	832
	Stem	1560	1046
	Whole plant	1245	995
Maize	Leaves	390	195
	Stem	598	420
	Whole plant	564	405
Napier bajra	Leaves	472	306
	Stem	915	635
	Whole plant	748	525

Guinea grass samples taken from four cuttings revealed significant differences in the nitrate content amongst cuttings [Table 4]. The nitrate content was lowest in fourth cutting and highest in second cutting. The reason for highest nitrate level in second cutting could be plentiful growth of crop and use of fertilizer (urea) in the fields after first cutting by the farmers. Four farmers applied urea after third cutting which was reflected as exceptionally high nitrate content (1705, 1525, 1678 and 2005 ppm) in fourth cutting plants collected from those fields. The data suggested that nitrate content decreases with the maturity of plant and use of nitrate containing fertilizers directly affects the nitrate accumulation in plants.

**Table 4:** Nitrate content in different cuttings of guinea grass (*n* = 20)

Number of cuttings	Nitrate content (ppm)
1^st^	580–695
2^nd^	1560–2090
3^rd^	1200–1260
4^th^	870–1005

The concentration of nitrate differs with the parts of plants when accumulation occurs. To determine the distribution of nitrate in the plants, the nitrate content was determined in leaves, stem and whole plant of berseem, bajra, maize, oats and toriya [Table 3]. Results showed the variability in nitrate level in different parts of plants. All forages exhibited similar results of having higher levels of nitrate in stem than in leaves. The difference of nitrate levels in stem and leaves was highly significant in maize and bajra, followed by berseem, oats and toriya. This might be due to differences in the ability of roots to take up nitrogen from the soil. These findings have confirmed that plant parts vary in nitrate content; parts close to the ground contain more nitrates and as we go higher along the length of plant, nitrate content goes on decreasing. Roots and stems have more nitrate content, followed by leaves,[3] whereas flowers and grains usually contain little or no nitrate.[9]

To study the effect of use of nitrate containing fertilizers on the nitrate accumulation in plants, the samples of berseem, bajra, maize, oats, sorghum and toriya were harvested from fields of Punjab Agricultural University, Ludhiana, where no chemical fertilizer was used. The mean values of nitrate content obtained in those fodder samples are shown in Table 1. Environmental factors contribute to variation in nitrate level in plants; however, in this study the effect of environmental factors was neutralized, as climatic conditions were the same for the samples collected from the villages and university fields. The samples collected on the same day from both the places were included in this study to minimize the effect of environmental factors. The nitrate levels of university field forages were lower than that of the field samples and were quite below the maximum safe limit (2500 ppm). The trend was same in all seasons (summer, winter, spring and rainy season). This indicated excessive use of urea in the village fields by the farmers to have a better yield. There is an urgent need to determine the soil nitrate content throughout India and to educate the ignorant farmers regarding the consequences of this excessive use of fertilizers.

Besides the above factors, weather conditions influence nitrate accumulation in plants significantly. Unfavorable weather conditions for plant growth, viz., drought, frost, extreme cold and cloudy weather, may increase nitrate accumulation in plants.[4] In the present study, nitrate level was significantly higher in forages when determined in adverse growing conditions, viz., cloudy, cold with frost, wet in winters and very hot drought conditions in summer compared to nitrate concentrations found in same fields under normal weather conditions. This is one of the reasons why cases of nitrate poisoning in Punjab are frequently encountered during the months of December and January when crops are affected by extreme cold and frost.[10] In very hot summer, the nitrification of bacteria in soil increases many folds which leads to nitrate accumulation in fodder. The cold and cloudy weather decreases the nitrate reductase activity and hence increases nitrate accumulation by plants. The conditions detrimental for plant growth including frost enhance accumulation of nitrate by reducing the surface area of the plant available for evaporation and photosynthesis.[9] Two outbreaks of nitrate poisoning in cattle due to Jumbo grass (sorghum hybrid) were reported in New Zealand. In both the incidents, the climatic factors were responsible for increasing nitrate content in forage. Accidental rain after a long dry summer, causing very rapid growth of grass, was a favorable condition for nitrate accumulation.[11] Similarly, sudden deaths due to nitrate poisoning occurred in cattle grazing ryegrass pasture for 6 hours in Australia. The quantitative analysis of ryegrass showed levels of 12.5% nitrate/nitrite on dry matter basis. The peculiar weather conditions, viz., summer, newly sown pasture, regenerated lush grass and cloudy environment, contributed to high nitrate levels in the grazed pasture.[12] A very cold, wet and windy weather contributed to another nitrate poisoning in the cattle fed sudax hay (*Sorghum* sp.) in South Wales, Australia. The toxicological laboratory determined the nitrate levels in hay to be up to 3.1%.[13]

Soil type also plays an important role in nitrate accumulation. The plants grown in acidic and phosphorus deficient soils are known to have greater nitrate content. The uptake of NO_3_^-^ is largely increased at slightly acid pH levels because of the higher H^+^ gradient across the plasma membrane at low pH, and possibly because increased H^+^ influx reduces the membrane potential and facilitates NO_3_^-^ uptake.[14] Crops take up and assimilate nitrogen as NH_4_^+^, NO_3_^-^ and various soluble organic compounds such as urea (CO(NH_2_)_2_) and amino acids.[2] In aerated soils with a pH above 4, NO_3_^-^ is the prevailing N compound and NH_4_^+^ is found only in low concentrations; but in waterlogged soils, the ratio of NO_3_^-^ and NH_4_^+^ is reversed mainly as a consequence of depressed bacterial nitrification activity and denitrification of NO_3_^-^.[14]

It is concluded that all plants contain nitrates but levels vary with the species, stage of growth, parts of plants and intrinsic ability to accumulate nitrate. Grasses are more sensitive to nitrate toxicity than cereals and pulses.[8] Addition of nitrate containing fertilizer should be avoided in the crops that tend to accumulate nitrate, viz., sorghum, oats, toriya, and if need arises, the quantity should be strictly adhered to the recommendations of agriculturists. The soil should be checked for its type, pH and nitrate levels before sowing the forages. In fields having higher nitrate levels, lower part of the crop near the ground should not be used as fodder because roots and stem store more nitrate than leaves. However, fodder can be used in the form of silage as ensiled forages contain less nitrate than fresh plants, but hay usually contains the same amount of nitrate as present in the fodder.[3] The forage containing high nitrate content (sorghum, oats and toriya) may be used by mixing with other crops having a low amount of nitrate to reduce the chances of toxicity in dairy animals. The forages should not be grown in the fields receiving sewage water or sludge or industrial effluents which may lead to serious nitrate toxicosis in the farm animals. Besides high levels of nitrate, other commonly used chemicals (pesticides, other fertilizers, etc.) in agricultural practices also occur in the environment. Synergistic or additive effect of these chemicals needs to be determined to minimize the risk of adverse effects on human and animal health.

Given it a multi-factorial problem, there is an urgent need to take on further research on the nitrate toxicity due to fodder crops in livestock using integrated approach involving different disciplines. The known information gathered from existing literature put forward many serious questions on man-made global nitrate pollution endangering the health and survival of man, livestock and wildlife population if agricultural and urban development continues without the incorporation of strict regulations to reduce the impact of nitrate on our environment. The current study may serve as background information to conduct further research for the better understanding of factors contributing to serious potential of nitrate accumulation in forages leading to animal toxicity.

## References

[1] Malhi SS, Girl KS, McCartney DH, Malmgren R (2004). Fertilizer management of forage crops in the Canadian Great Plains. Recent Res Dev Crop Sci.

[2] Falkengren-Grerup U, Mansson KF, Olsson MO (2004). Uptake capacity of amino acids by ten grasses and forages in relation to soil acidity and nitrogen availability. Environ Exp Biol.

[3] Radiositis OM, Gay CC, Blood DC, Hinchcliff KW (2000). Veterinary Medicine.

[4] Kahn CM (2005). Toxicology: Nitrate and nitrite poisoning. The Merck Veterinary Manual.

[5] Catalado DA, Haroon M, Schrader LE, Youngs VC (1975). Rapid colorimetric determination of nitrate in plant, tissues by titration of salicylic acid. Commun Soil Sci Plant Anal.

[6] Burrows GE, Tyrl RJ (1989). Plants causing sudden death in livestock. Vet Clin North Am Food Anim Pract.

[7] Richards SA, McCarty TR, Lawrence JH Prevention and control of nitrate toxicity in cattle. http://www.extension.org/pages/prevention_andcontrol_of_nitrate_Toxicity_in_cattle.

[8] Bose MS (1996). Nitrate toxicity in fodder crop and varietal variabilities. Madras Agril J.

[9] Osweiler GD (1996). Toxicology.

[10] Annual Progress Report of Project Directorate on Animal Disease Monitoring and Surveillance, 2003/2004, Submitted by Department of Epidemiology and Preventive Veterinary Medicine.

[11] Vermunt J, Visser R (1987). Nitrate toxicity in cattle. New Zealand Vet J.

[12] Nicholls TJ (1980). Nitrate/nitrite poisoning of cattle on ryegrass pasture. Australian Vet J.

[13] Carrigan MJ, Gardner IA (1982). Nitrate poisoning in cattle fed Sudax (Sorghum sp.hybrid) hay. Aust Vet J.

[14] Vessey JK, Henry LT, Chaillou S, Raper CD (1990). Root-Zone acidity affects relative uptake of nitrate and ammonium from mixed nitrogen sources. J Plant Nutr.

